# Dissociating reading and auditory comprehension in persons with aphasia

**DOI:** 10.1093/braincomms/fcae102

**Published:** 2024-03-25

**Authors:** Rachael M Harrington, Sigfus Kristinsson, Janina Wilmskoetter, Natalie Busby, Dirk den Ouden, Chris Rorden, Julius Fridriksson, Leonardo Bonilha

**Affiliations:** Department of Communication Sciences and Disorders and Center for Research on the Challenges of Acquiring Language and Literacy, Georgia State University, Atlanta, GA 30310, USA; Department of Communication Sciences and Disorders, University of South Carolina, Columbia, SC 29208, USA; Department of Health and Rehabilitation Sciences, Medical University of South Carolina, Charleston, SC 29464, USA; Department of Communication Sciences and Disorders, University of South Carolina, Columbia, SC 29208, USA; Department of Communication Sciences and Disorders, University of South Carolina, Columbia, SC 29208, USA; Department of Psychology, University of South Carolina, Columbia, SC 29208, USA; Department of Communication Sciences and Disorders, University of South Carolina, Columbia, SC 29208, USA; School of Medicine Columbia, University of South Carolina, Columbia, SC 29208, USA

**Keywords:** aphasia, alexia, reading, lesion-symptom mapping, stroke

## Abstract

Language comprehension is often affected in individuals with post-stroke aphasia. However, deficits in auditory comprehension are not fully correlated with deficits in reading comprehension and the mechanisms underlying this dissociation remain unclear. This distinction is important for understanding language mechanisms, predicting long-term impairments and future development of treatment interventions. Using comprehensive auditory and reading measures from a large cohort of individuals with aphasia, we evaluated the relationship between aphasia type and reading comprehension impairments, the relationship between auditory versus reading comprehension deficits and the crucial neuroanatomy supporting the dissociation between post-stroke reading and auditory deficits. Scores from the Western Aphasia Battery—Revised from 70 participants with aphasia after a left-hemisphere stroke were utilized to evaluate both reading and auditory comprehension of linguistically equivalent stimuli. Repeated-measures and univariate ANOVA were used to assess the relationship between auditory comprehension and aphasia types and correlations were employed to test the relationship between reading and auditory comprehension deficits. Lesion-symptom mapping was used to determine the dissociation of crucial brain structures supporting reading comprehension deficits controlling for auditory deficits and vice versa. Participants with Broca’s or global aphasia had the worst performance on reading comprehension. Auditory comprehension explained 26% of the variance in reading comprehension for sentence completion and 44% for following sequential commands. Controlling for auditory comprehension, worse reading comprehension performance was independently associated with damage to the inferior temporal gyrus, fusiform gyrus, posterior inferior temporal gyrus, inferior occipital gyrus, lingual gyrus and posterior thalamic radiation. Auditory and reading comprehension are only partly correlated in aphasia. Reading is an integral part of daily life and directly associated with quality of life and functional outcomes. This study demonstrated that reading performance is directly related to lesioned areas in the boundaries between visual association regions and ventral stream language areas. This behavioural and neuroanatomical dissociation provides information about the neurobiology of language and mechanisms for potential future treatment interventions.

## Teaser

Harrington *et al*. report that auditory and reading comprehension can be dissociated in persons with chronic aphasia. Auditory comprehension explains <50% of the variance in performance on reading comprehension tasks. Reading comprehension deficits are directly linked to lesioned areas between visual association regions and ventral stream language areas.

## Graphical Abstract

**Graphical Abstract:**
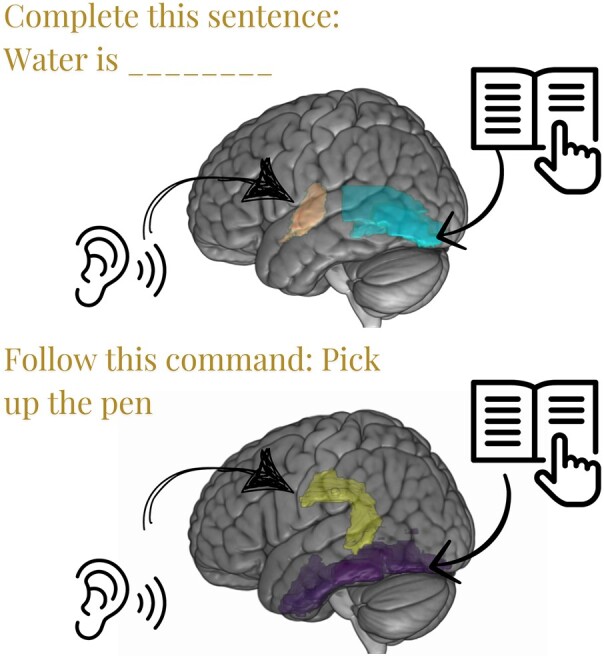

## Introduction

The ability to read is functionally intertwined with perceptual and linguistic abilities.^1^ To comprehend a written word, readers must be able to visually perceive the word, decode the word and determine its meaning within an existing lexicon.^2^ From a developmental perspective, the visual network and the language networks develop earlier than the reading network.^3-7^ From an evolutionary perspective, reading is a later developing process with the first record of a written alphabet occurring only 3000 years ago.^8^

The reading system is believed to depend on neuroanatomical networks composed of associative visual areas in the occipitotemporal region, a sublexical or phonological processing route associated with the dorsal stream in the temporoparietal region and a lexical or semantic processing route associated with the ventral stream in the frontotemporal region.^2,9,10^ Among children and adults with typical reading skills and developmental dyslexia, decreased activation and connectivity between and within these regions, specifically in the temporoparietal region, are associated with worse reading outcomes.^11-16^ Stroke lesions within this network also cause reading deficits frequently associated with aphasia. These reading deficits are often collectively referred to as alexia, an acquired reading impairment where lesions within dorsal or ventral streams correspond with phonological or semantic reading deficits, respectively.^1,17-19^ While alexia can be further subdivided into subtypes, this study will not parse the specific types of alexia.

Post-stroke reading and language impairment offer potential insight into the relationship between reading and language networks. Reading impairment co-occurs with language impairment in 68–80% of persons with aphasia.^20,21^ This could be related to the large lesions associated with aphasia that may overlap with regions specifically related with reading, similar to the 80% co-occurrence of hemiplegia in persons with Broca’s aphasia.^22^ However, and more likely, there are shared language processes that are common to reading and auditory communication. In fact, language and reading networks often involve indistinguishable regions when measured with task-functional MRI (fMRI).^1,23-25^ While reading comprehension cannot occur independently of language processing, reading is at least partially dissociable from a clinical perspective. Apart from the early processing step of auditory versus visual input, reading can be dissociated from language processing neurobiologically, relying on different pathways and networks, and functionally. Parietal and temporal lobe activations differ distinctly between typical auditory and reading comprehension and may help explain the variability seen in post-stroke reading and auditory comprehension deficit severity.^26,27^ Since post-stroke reading impairment can be present without auditory language impairments, this dissociation can be leveraged to distinguish critical behavioural deficits and supporting neuroanatomical networks related to auditory versus reading communication, and ultimately be used for prognosis and the development of treatment interventions.

Several studies have investigated the neurobiology of post-stroke reading impairment and of different aspects of reading function.^1,18,19,28-35^ Few, however, have examined the relationship between aphasia and reading impairment. A clear causal relationship between aphasia type and long-term impairment in reading comprehension would help guide clinical intervention choices early in treatment. Cherney^36^ provided broad categorization of the relationships between alexia and aphasia subtypes based on case studies, stating that while there is no defined relationship between aphasia type and alexia, deep alexia is often present in Broca’s aphasia, semantic alexia frequently accompanies transcortical sensory aphasia and phonological alexia can co-occur with anomic aphasia.

Brookshire *et al*.^20^ briefly explored the relationship between aphasia type and oral reading accuracy. Among patients with co-occurring aphasia and alexia, they observed that persons with anomic and transcortical motor (TCM) aphasia had better oral reading for regular and irregular words than persons with Wernicke’s, Broca’s or global aphasia.^20^ There are, however, limited available data to form a predictable relationship between aphasia type and alexia syndrome despite clear delineations of lesion locations associated with aphasia and alexia.^36-38^ Importantly, the crucial anatomy supporting reading deficits in aphasia have not been directly examined with lesion-based studies while controlling for auditory comprehension. Functional imaging studies can illustrate the networks related to reading, but whether these are crucial for behavioural performance can only be defined by lesion-related impairments.^39^ Moreover, the clinical implications of the dissociation between reading and auditory impairments are self-evident since rehabilitation strategies for communication improvement could target functional impairments or potentially leverage neuroanatomical information to guide treatment planning. If clinical reading deficits can be dissociated via distinct neurobiology, then written and read communication can potentially be targeted to improve participation in this crucial aspect of quality of life.

We hypothesized that reading comprehension deficits in aphasia would be partly, but not fully, explained by auditory comprehension deficits. We also hypothesized that unique neuroanatomical areas would be linked with reading comprehension deficits when accounting for auditory comprehension, apart from the initial modality-specific processes (visual and auditory input pathways). We anticipated that reading-specific regions would be situated at the intersection of visual association areas and language comprehension regions. Since reading comprehension is postulated to depend more directly on semantic association processes, we postulated that critical regions for reading would be primarily located in the posterior boundaries of the ventral stream.^40^ To investigate these hypotheses, we evaluated a large cross-sectional dataset of 70 persons with chronic post-stroke aphasia with comprehensive linguistically matched reading and auditory comprehension tests. We examined linguistic features in relationship with aphasia types, and a lesion-based approach was used to assess the multivariate relationship between the location of the stroke lesion and reading comprehension controlling for auditory comprehension and vice versa.

## Materials and methods

Behavioural and imaging data from participants (*N* = 70) with chronic language impairment (aphasia) after left-hemisphere stroke were retrospectively analysed. All participants were part of the Predicting Outcomes of Language Rehabilitation (POLAR; NCT03416738) study at the Center for the Study of Aphasia Recovery (C-STAR) at the University of South Carolina and the Medical University of South Carolina.^41,42^ ASHA-certified speech-language pathologists with experience working with individuals with aphasia administered all assessments.

The following inclusion/exclusion criteria were applied: chronic (>12 months post-onset) left-hemisphere ischaemic or haemorrhagic stroke, between 21 and 80 years of age and English as their primary language for at least 20 years. Participants were excluded if they had bilateral lesions, severely limited speech output [Western Aphasia Battery—Revised (WAB-R) Spontaneous Speech rating scale score of 0–1] or auditory comprehension (WAB-R Auditory Comprehension Score of 0–1), were contraindicated for MRI or were unable to provide written or verbal consent. Individuals with multiple strokes were eligible if all lesions were confined to the left supratentorial territory. Eight individuals without aphasia after left-hemisphere stroke were included in the analysis. These participants provide more variability for lesion analysis and provide a degree of control. Behavioural variability allows for assessing the range with which lesions are associated with impairment. Participants over the age of 80 were excluded, to control for effects of normal aging and to ensure comfort during lengthy MRI sessions. English as a primary language for at least 20 years ensured fluency in English. Patients with severely limited speech output were excluded as these data were collected as part of a larger clinical trial and these participants had a lower probability of improvement. Inclusion and exclusion criteria are explained in further detail in publications specific to the clinical trial.^41,42^ Data were collected between August 2016 and August 2020. This study was approved by the Institutional Review Boards at the University of South Carolina and the Medical University of South Carolina. All participants provided written informed consent.

**Figure 1:**
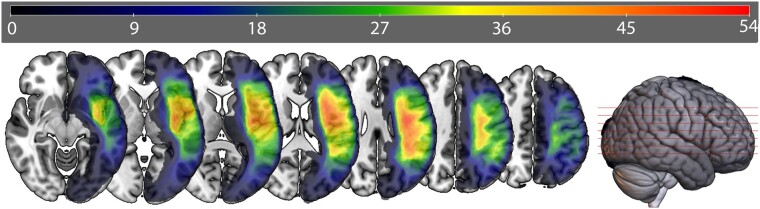
**Lesion overlap map: lesion overlap map of hand-drawn lesions for all participants.** Colours represent the number of participants with lesions in each voxel (max overlap = 54).

Aphasia subtype was determined based on the WAB-R subscores following published norms. Classifications were spread across several types of aphasia, specifically anomic (*n* = 13), Broca’s (*n* = 31), conduction (*n* = 10), global (*n* = 4), TCM (*n* = 1), Wernicke’s (*n* = 3) and not aphasic (*n* = 8). Mean WAB-R score was 61.9 (SD = 25.8). Mean lesion volume was 119.90 mm^3^ (SD = 82.72 mm^3^). Lesion overlap maps for all participants are shown in Fig. 1 and demographics in Table 1.

**Table 1:** Descriptive statistics of demographic variables

Demographic variables (*N* = 70)	Mean (SD)/count
Test age	61.14 (10.66)
Education	15.56 (2.39)
Months post-stroke	54.53 (53.00)
Stroke age	56.57 (11.50)
Lesion volume (cubic millimetres)	119.90 (82.72)
Sex (males:females)	43:27
Handedness (left:right)	5:65
WAB AQ^a^	62.05 (25.76)
WAIS matrices subtest	11.97 (5.77)
Stroke type (ischaemic:haemorrhagic:other)	48:20:2
Aphasia types (count)	
Anomia	13
Broca’s	31
Conduction	10
Global	4
Transcortical sensory	1
Wernicke’s	3
None	8

^*a*^WAB Aphasia Quotient (AQ).

### Behavioural measures

#### WAB-R and WAB-R reading supplement

All participants completed the WAB-R and the first two subtests of the WAB-R reading supplement.^43^ The WAB-R is one of the most frequently used assessments of aphasia type and severity. It has high levels of criterion validity, inter- and intra-rater reliability, test–retest reliability, convergent validity and discriminant validity.^43^

The WAB-R contains a main test consisting of four sections with 10 tasks and a reading supplement consisting of nine tasks. The four sections of the main test consist of spontaneous speech, auditory comprehension, repetition and naming and word finding. The reading supplement, while less used, also has strong reliability and validity. The purpose of the reading section is to ‘measure a patient’s oral reading ability and his or her reading comprehension of words and sentences.’ The reading supplement has high test–retest reliability,^44^ and the reading subtests correlated highly with the Neurosensory Center for Comprehensive Examination of Aphasia during initial validation.^45^

#### Auditory and reading sentence completion

Two subtests on the WAB-R and the WAB-R reading supplement ask participants to complete a sentence. In the sentence completion [Auditory Sentence Completion (A-SC)] subtest of the WAB-R, participants are asked to orally complete five sentences spoken by the examiner for a maximum score of 10. Examiners state ‘Complete what I say. For example, ice is ____.’ A similar task is present in the WAB-R reading supplement. In the reading comprehension of sentences [Written Sentence Completion (R-SC)] subtest, participants are asked to read a sentence aloud or silently and choose the best word to complete eight sentences from four options with semantic and phonological foils for a maximum score of 40. Examiner’s state ‘read this sentence and point to the missing word. Choose the best word from these.’

These two subtests are paired in this analysis as measures of auditory and reading sentence completion. Because base stimuli were different, no carry-over effects are expected. Both tasks require semantic and syntactic comprehension with either a verbal or nonverbal response, but A-SC requires auditory comprehension while R-SC requires orthographic comprehension. Sentence completion is a validated measure of reading comprehension.^46^ Subtest scores are analysed as per cent of the total score.

#### Auditory and reading sequential commands

Two subtests on the WAB-R and the WAB-R reading supplement ask participants to follow a series of sequential commands. In the sequential commands [Auditory Following Commands (A-FC)] subtest of the WAB-R, patients are asked to follow one-step to multi-step commands by performing physical actions. The subtest consists of 11 commands for a maximum score of 80. The examiner states ‘I am going to ask you to do some things.’ In the reading commands [Written Following Commands (R-FC)] subtest of the WAB-R, patients are asked to read a sentence aloud and perform the actions in the sentence. The test consists of six sentences for a maximum score of 20. The examiner states ‘I want you to read this aloud and then do what it says.’

Likewise, these two subtests are paired in this analysis as measures of auditory and reading comprehension of commands. No carry-over effects are expected between subtests. This task pair has the benefit of the same nonverbal task execution without the confound of a verbal response. This task pair is also a measure of functional comprehension and reading, or the comprehension and reading abilities needed to perform an everyday task.^47^ Subtest scores are analysed as per cent of the total score.

### Neuroimaging

#### Neuroimaging acquisition and preprocessing

High resolution T_1_- and T_2_-weighted neuroimaging data were collected on a Siemens Trio 3T scanner equipped with a 12-channel (Trio configuration) or 20-channel (following upfit to Prisma configuration) head coil using the following parameters: T_1_-weighted imaging utilized an MP-RAGE sequence with 1 mm isotropic voxels, a 256 × 256 matrix size, a 9° flip angle, and a 92-slice sequence with repetition time (TR) = 2250 ms, inversion time (TI) = 925 ms and echo time (TE) = 4.11 ms. T_2_-weighted scans were acquired using the same angulation and volume centre as the T_1_ scan. This 3D T_2_-weighted SPACE sequence used a resolution of 1 mm^3^ was used with a field of view = 256 × 256 mm, 160 sagittal slices, variable degree flip angle, TR = 3200 ms, TE = 212 ms and ×2 GRAPPA acceleration (80 reference lines).

#### Lesion segmentation

Lesions were drawn onto T_2_-weighted images in native space. Lesions were traced by either an expert neurologist or trained study staff in MRIcron.^39^ Tracings were performed blind to behavioural and demographic data. Each participant’s T_2_ image was co-registered to their T_1_ image, and binary lesion maps were then spatially transformed into native T_1_ space using the resulting function. Resliced lesion maps were smoothed with a 3 mm full-width half maximum Gaussian kernel to remove sharp edges associated with hand drawing. Enantiomorphic segmentation–normalization was then employed using SPM12 and a series of custom, publicly available MATLAB scripts (nii_preprocess; www.nitrc.org/projects/niistat).^48^ This included creation of a mirrored image of the right hemisphere, which was co-registered to the native T_1_ image. A chimeric image was then created, based on the native T_1_ scan with the lesioned tissue replaced by tissue from the mirrored hemisphere. SPM12’s unified segmentation–normalization warped this chimeric image to standard space, and the resulting spatial transform was applied to the native T_1_ scan as well as the lesion map and the T_2_/DWI image.

#### Lesion-symptom mapping

To identify lesion locations associated with poor reading comprehension in persons with aphasia, we performed univariate lesion-symptom mapping (LSM) using the NiiStat Toolbox. This toolbox has previously been used to analyse neuroimaging data in persons with aphasia.^49-54^ Univariate analyses were run on two sets of behavioural data (A-SC versus R-SC and A-FC versus R-FC) with normalized lesion maps and compared to regions of interest (ROIs) in the Johns Hopkins University grey and white matter atlas (JHU) controlling for lesion volume.^55^ The analysis included all left-hemisphere ROIs within the JHU atlas (85 regions). Results reported are based on JHU ROIs. Univariate analyses were chosen because of the limited sensitivity of the available behavioural measures. While univariate analysis is more likely to be associated with false positive errors, it is less likely to result in false negative errors.^56-58^ Due to the limited sensitivity of our behavioural measures, we chose to prioritize avoiding false negative errors, notwithstanding, the univariate method was applied with a conservative correction for multiple comparisons (permutation testing at 3000).

### Statistical analysis

#### Demographic data

Descriptive statistics (mean and standard deviation) for age, lesion volume and WAB-R Aphasia Quotient (AQ) were calculated in IBM SPSS Statistics (version 26, released 2019, IBM Corp).

#### Behavioural analysis

To normalize different maximum scores between subtests, WAB-R and WAB-R reading supplement raw subtest scores were normalized to a percentage based on total possible score. Normalized scores were entered into a repeated-measures ANOVA. Normalized R-SC and R-FC subtest scores served as the repeated within-subject measure and WAB-R aphasia classification as the between-subjects variable (*α* = 0.05) in SPSS version 26.

Additionally, two separate univariate ANOVAs were performed on the normalized R-SC and R-FC subtest scores. These analyses specifically examined the effects of the WAB-R aphasia classification as the independent variable, maintaining a significance level of *α* = 0.05. This approach allowed for a comprehensive exploration of the relationships between aphasia classification, task types, and their interaction on performance, providing insights into the nuances of participants’ abilities across tasks and aphasia types.

To obtain the strength and direction of the relationships between auditory and written subtests, normalized subtest scores were correlated in R version 4.1.3 using the formula:

cor.test(Auditory Comprehension Measure∼Visual  Comprehension Measure).

Analyses were run on two comparisons (A-SC versus R-SC and A-FC versus R-FC). All figures were created using the ggplot2 library in R.^59^

#### LSM analysis

Two lesion-mapping analyses were performed within the NiiStat GUI (A-SC versus R-SC and A-FC versus R-FC). Lesion analyses were performed using Freedman–Lane permutation thresholding (3000 permutations) so that each behaviour was used as a nuisance regressor against other behaviours.^60^ More specifically, we assessed R-SC performance controlling for A-SC and vice versa, and R-FC controlling for A-FC and vice versa. Analyses were performed with a corrected *P*-value of *P* < 0.05 and correction for multiple comparisons using Freedman–Lane permutation thresholding.

## Results

### Relationship between aphasia classification and reading comprehension

**Figure 2:**
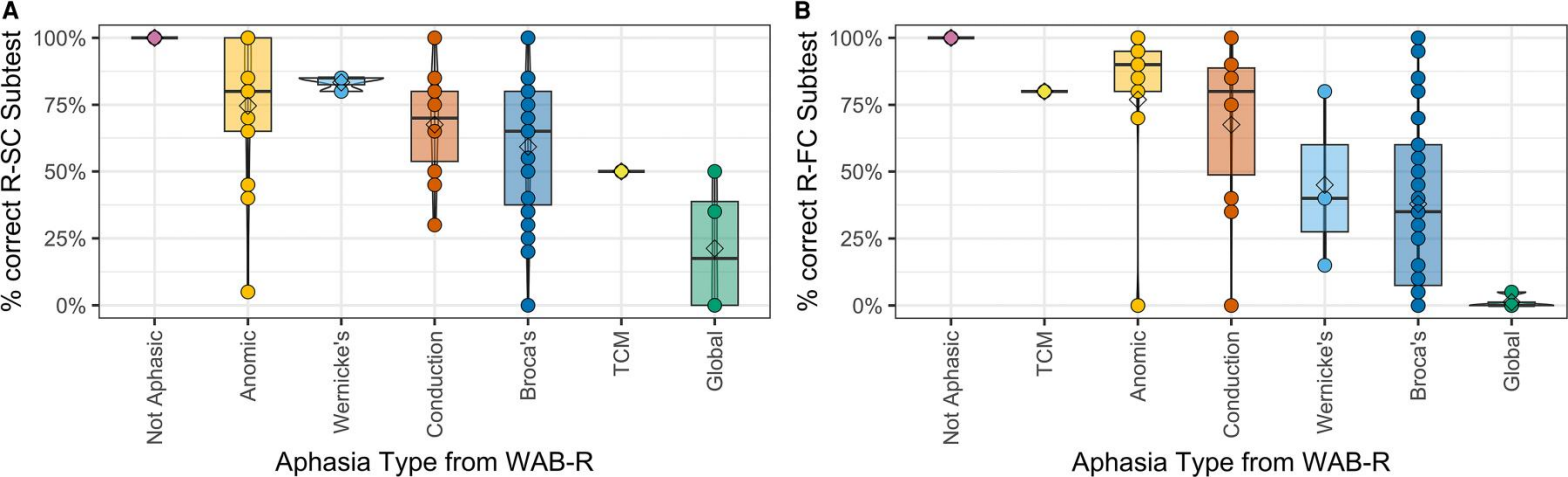
**Reading performance by aphasia type: reading performance by aphasia type for 70 participants with language impairment [anomic (*n* = 13), Broca’s (*n* = 31), conduction (*n* = 10), global (*n* = 4), TCM (*n* = 1), Wernicke’s (*n* = 3) and not aphasic (*n* = 8)].** (**A**) Performance on the comprehension of sentences (R-SC) subtest of the WAB-R supplemental section (global < anomic: *t* = −4, *P* < 0.01; global < conduction: *t* = −3.2, *P* < 0.05; global < not aphasic: *t* = −5.2, *P* < 0.001; global < Wernicke’s: *t* = −3.3, *P* < 0.05; not aphasic > Broca’s: *t* = 4, *P* < 0.01). (**B**) Performance on the following reading commands (R-FC) subtest of the WAB-R supplemental section (global < anomic: *t* = −4.4, *P* < 0.001; global < conduction: *t* = −3.6, *P* < 0.01; global < not aphasic: *t* = −5.3, *P* < 0.001; not aphasic > Broca’s: *t* = 5.2, *P* < 0.001). Participants classified with global aphasia (0–20% accuracy) or not aphasic (100% accuracy) differed from most other aphasia classifications, but no other aphasia classifications were significantly different.

To explore the relationship between reading comprehension and aphasia subtype, a repeated-measures ANOVA was performed for the normalized reading comprehension measures (R-SC and R-FC). Our sample represented a range of aphasia subtypes: anomic (*n* = 13), Broca’s (*n* = 31), conduction (*n* = 10), global (*n* = 4), TCM (*n* = 1), Wernicke’s (*n* = 3) and not aphasic (*n* = 8). The main effect of the reading tasks *F*(1,63) = 2.24, *P* = 0.14 did not reach statistical significance. The main effect of aphasia type *F*(6,63) = 7.97, *P* < 0.001 and the interaction effect between reading tasks and aphasia type *F*(6,63) = 3.84, *P* < 0.005 were statistically significant. In *post hoc* univariate ANOVAs, persons classified as not aphasic or with anomic or conduction aphasia had the highest performance while persons classified with Broca’s or global aphasia had the worst performance on both measures of reading comprehension. Participants with Wernicke’s aphasia performed better on written sentence completion than written following commands. Only a single participant was classified with TCM aphasia, and this participant performed better on written following commands than written sentence completion. Within-group variability, however, was high for participants with Broca’s, anomic, conduction and Wernicke’s aphasia. As a result, the only significant differences were between those with a classification of not aphasic (R-SC: 100% accuracy, R-FC: 100% accuracy) or with global aphasia (R-SC: 0–50% accuracy, R-FC: 0–5% accuracy). For this analysis, we have focused on syndrome-based classification but in the below analyses and future analyses, we will focus on symptom-based classification, which is preferred.^61^ See Fig. 2.

### Relationship between auditory and reading comprehension

To better understand the relationship between auditory and reading comprehension, we correlated scores from the paired subtests (A-SC and R-SC; A-FC and R-FC).

#### Sentence completion

**Figure 3:**
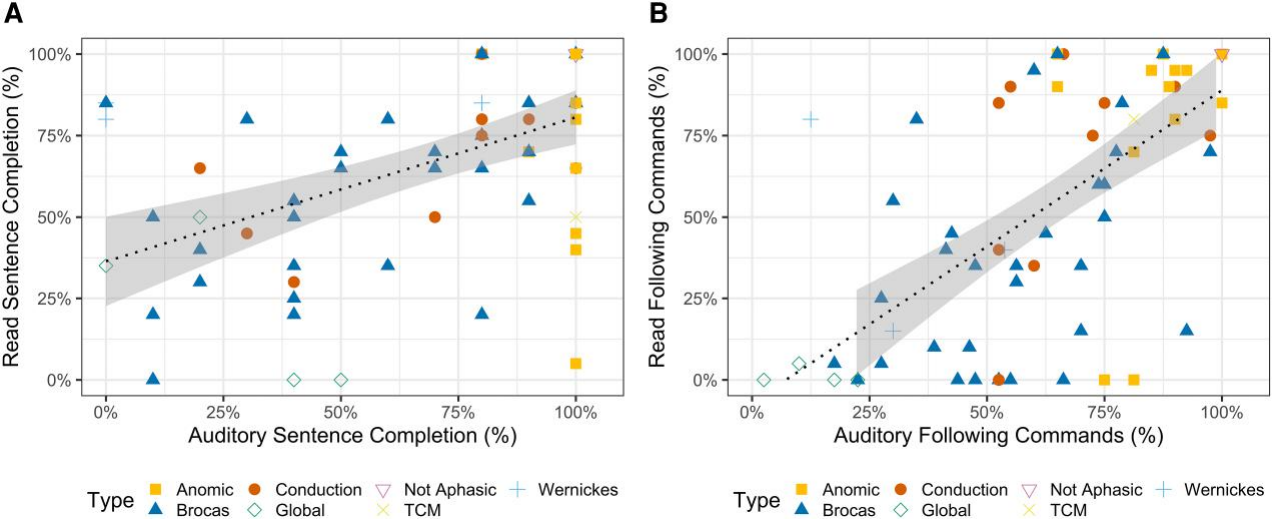
**Individual task performance: individual comparison of successful performance on reading versus auditory comprehension tasks.** Type of aphasia marked by colour. (**A**) Performance on the comprehension of reading sentences subtest of the WAB-R supplemental section (*r*^2^ = 0.26, *P* < 0.001). (**B**) Performance on the following reading commands subtest of the WAB-R supplemental section (*r*^2^ = 0.44, *P* < 0.001).

Normalized A-SC and R-SC subtest scores were correlated. We found a significant positive correlation between A-SC and R-SC (*r* = 0.5, *P* < 0.001), and auditory comprehension explained 26% of the variance in reading comprehension. See Fig. 3A.

#### Following sequential commands

Normalized A-FC and R-FC subtest scores were correlated. We found a significant positive relationship between A-FC and R-FC (*r* = 0.6, *P* < 0.001). Auditory comprehension explained 44% of the variance in reading comprehension. See Fig. 3B.

### LSM to differentiate auditory and reading comprehension

#### Sentence completion

**Figure 4:**
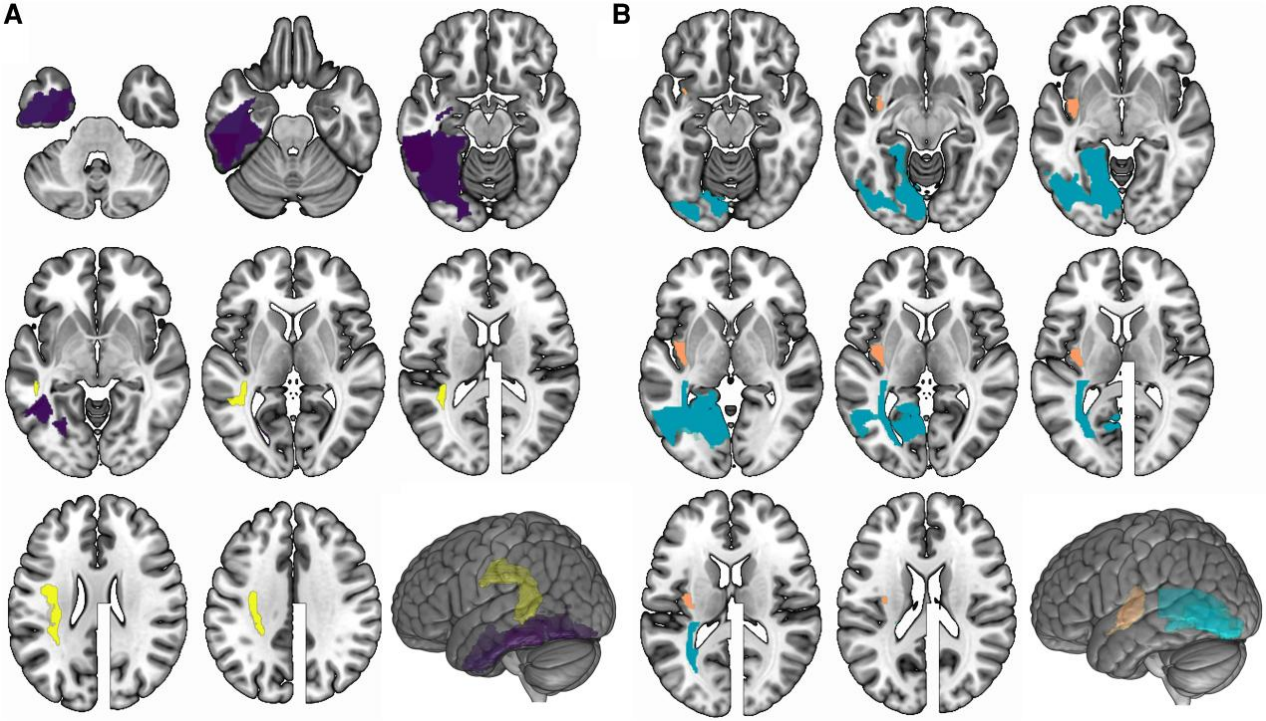
**Lesion location and task performance: lesion locations associated with auditory comprehension and reading comprehension in participants with language impairment.** Coloured regions show region of interest in the Johns Hopkins University atlas that survived statistical testing with *P* < 0.05 controlling for lesion volume. (**A**) Relationship between lesion location and performance on the sentence completion (A-SC) task of the WAB-R and the comprehension of written sentences (R-SC) subtest of the WAB-R supplemental section. Worse auditory comprehension (yellow): superior longitudinal fasciculus (*Z* = −3.36). Worse reading comprehension (purple): inferior temporal gyrus (*Z* = −3.34), fusiform gyrus (*Z* = −3.43) and posterior inferior temporal gyrus (*Z* = −3.56) (**B**) Relationship between lesion location and performance on the comprehension of sequential commands (A-FC) subtest of the WAB-R and the following reading commands subtest (R-FC) of the WAB-R supplemental section. Worse auditory comprehension (orange): posterior insula (*Z* = −4.0). Worse reading comprehension (blue): inferior occipital gyrus (*Z* = −3.3), lingual gyrus (*Z* = −3.3) and posterior thalamic radiation (*Z* = −3.31).

The LSM analysis identified several regions in the JHU atlas within the left hemisphere associated with worse reading and auditory comprehension in the sentence completion subtests. Worse auditory comprehension performance was associated with damage to the superior longitudinal fasciculus (*Z* = −3.36). Worse reading comprehension performance was associated with damage to the inferior temporal gyrus (*Z* = −3.34), fusiform gyrus (*Z* = −3.43) and posterior inferior temporal gyrus (*Z* = −3.56) (Fig. 4).

#### Following sequential commands

The LSM analysis identified several regions in the JHU atlas within the left hemisphere associated with worse reading and auditory comprehension in the following sequential commands subtests, Worse auditory comprehension performance was associated with damage to the posterior insula (*Z* = −4.0). Worse reading comprehension was associated with damage to the inferior occipital gyrus (*Z* = −3.3), lingual gyrus (*Z* = −3.3) and posterior thalamic radiation (*Z* = −3.31).

## Discussion

This is the first study to directly dissociate lesion correlates of auditory from reading comprehension deficits in persons with aphasia and to examine lesion correlates of both traditional sentence reading and functional reading, or reading associated with a specific task (following commands). Overall, our results indicated that traditional sentence comprehension reading deficits as well as reading specific tasks were more directly associated with aphasia severity than with aphasia type. Though there was an interaction effect between the reading tasks and aphasia type, *post hoc* analysis indicates that this is mostly driven by the presence of globally aphasic or not aphasic participants. This is an interesting finding that can be considered in agreement with the theoretical neuroanatomical framework of reading comprehension in general. More specifically, reading comprehension is hypothesized to engage primarily the ventral-semantic stream including the occipitotemporal, the middle temporal regions, but also supra-Sylvian regions such as the inferior frontal regions.^40^ These are areas commonly lesioned in Wernicke’s, anomic, global and Broca’s aphasia.^62^

As expected, this study also demonstrated that auditory comprehension predicted reading comprehension. This is somewhat expected given the shared semantic–lexical correlates between both tasks. However, the literature on this topic has shown mixed results on this dissociation. Some studies showed no differences in auditory and reading comprehension in patients with anterior and posterior alexia, but others found a clear dissociation between auditory and reading comprehension in persons with a mix of aphasia types.^63-65^ It should be noted that our study evaluated a much larger sample size compared with the existing studies in the literature, and we directly tested the relationship between linguistically similar behavioural tasks, where the auditory and the reading comprehension could be considered equivalent at a semantic and lexical level. We observed that auditory comprehension only partially explained the variance in reading comprehension for sentence completion and following sequential commands. These results suggest that individuals who have difficulty with auditory comprehension are likely to have difficulty with reading comprehension but that auditory comprehension deficits alone cannot explain the level of reading comprehension deficits.

The LSM analysis identified several regions within the left hemisphere associated with worse reading and auditory comprehension for both sentence completion and following sequential commands. Controlling for reading deficits, the regions directly associated with worse auditory comprehension were the superior longitudinal fasciculus for sentence completion and the posterior insula for following sequential commands. Based on models of auditory comprehension, these regions are most associated with long-range dorsal stream fibres between inferior frontal gyrus and posterior superior temporal gyrus related to syntactic relations and analysis of semantic and syntactic relations, respectively.^66^ These functions are also necessary for written comprehension; however, syntactic relations and analysis of semantic and syntactic relations may be associated with other regions in the written comprehension network (e.g. angular gyrus). Another possibility is that in controlling for auditory comprehension, regions that share function between the two comprehension networks may have stronger associations with one form of comprehension as opposed to the other based on sample characteristics. Given an analysis that does not control for one with the other, it is likely that we would get more shared and convergent results, especially later in the networks (e.g. frontal and anterior temporal regions).

Interestingly, a study of the same WAB-R sequential commands subtest found that worse auditory comprehension was associated with lesions in left posterior middle temporal gyrus.^27^ This result is consistent with other studies of lesion correlates of auditory comprehension.^26,67-71^ As the current study was designed to dissociate auditory from reading comprehension, it is possible that this is a shared region for both types of comprehension with posterior insula more specifically relating to auditory comprehension of sequential commands.

The regions associated with worse reading comprehension were the inferior temporal gyrus, fusiform gyrus and posterior inferior temporal gyrus for sentence completion, and the inferior occipital gyrus, lingual gyrus and posterior thalamic radiation for following sequential commands. These regions are posited to be involved in letter and word recognition, sight word recognition.^40^ These results are also consistent with the previous literature that associated surface dyslexia with left temporal lesions, specifically in the posterior middle temporal gyrus, posterior inferior temporal gyrus, posterior inferior insula, middle occipital gyrus, posterior superior temporal gyrus and optic radiation and associated sentence-level comprehension with left temperooccipital cortex and lateral temporal cortex with more anterior lesion locations in the temporal pole and adjacent to the insula.^17,72^

Several participants show a superiority of written language performance over auditory language performance. Interestingly, the three participants that showed superior performance on written sequential commands were different from the three participants that showed superior performance on written sentence completion, indicating that the differences do not solely lie in written over auditory performance. There were no consistent predictive patterns in age, gender or race. Participants with better reading comprehension during the sentence completion task were classified as having very severe or severe aphasia according to their WAB AQ (range: 22.8–33.9). The response selection from a set of possible answers in the written form of this task as opposed to the independent generation of response in the auditory form of the task may have benefited those with more severe aphasia. This may be further evidenced by performance comparisons between tasks. Participants who performed well on the written commands task also performed well on the written sentence completion task but good performance on the written sentence completion task did not translate to good performance on the written sequential commands task. The three participants who had higher written sentence completion scores only had between 15 and 40% accuracy on the written sequential commands task. Participants with superior reading comprehension during the sequential commands task were classified per WAB AQ with moderate aphasia (range: 54.8–69.2). Lesions were consistent with results as reported above. Participants who performed better on reading commands all had near total lesions in the posterior insula but no lesioned areas within lingual gyrus, inferior occipital gyrus or posterior thalamic radiation. Similarly, all three participants with better performance during completion of written sentences had near total lesions within the superior longitudinal fasciculus but no lesioned areas within the inferior temporal gyrus or fusiform gyrus.

**Figure 5:**
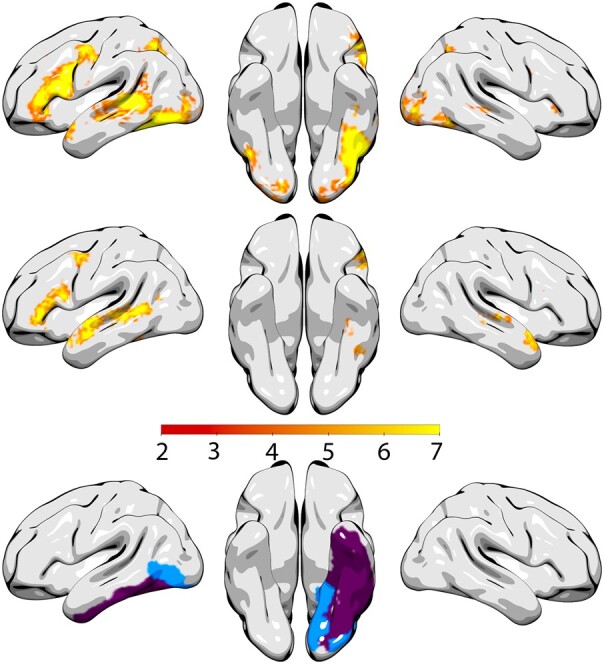
**Comparison with functional MRI assessments of reading: metanalytical functional MRI assessments of reading (*top* row) and sentence comprehension (*middle* row) demonstrate a high degree of overlap between active regions, with notable differences related to a higher engagement of inferior temporal and parietal regions during reading (images obtained from database available at Neurosynth.org version 0.7, released July, 2018, which includes 507 891 activations reported in 14 371 studies.** The terms displayed here are ‘reading’ and ‘sentence comprehension’ and the maps are association test *Z*-scores with false discovery rate (FDR) correction at *P* < 0.01. The lesion-symptom mapping results from this study, however, demonstrated that not all areas are necessary for reading when controlling for auditory comprehension. Those that are crucial for sentence comprehension include the inferior temporal gyrus and the fusiform gyrus (in purple), and the inferior occipital gyrus and the lingual gyrus (in blue) are crucial for reading commands (with the thalamic radiation not shown in this figure).

Interestingly, metanalytical evaluations of fMRI studies related to reading and auditory comprehension demonstrate a large degree on overlap between recruited regions (Fig. 5). Both tasks engage both dorsal and ventral perisylvian networks, with a noticeable difference related to a higher recruitment of ventral temporal and parietal regions during reading. However, based on fMRI alone, it is not possible to determine if these differences are related to crucial areas supporting reading, or simply involved in reading. In this study, we demonstrated that reading deficits, controlling for auditory impairments, are independently associated with damage to the ventral temporal regions, namely the inferior temporal gyrus (including its posterior aspect) and the fusiform gyrus for sentence completion, and the inferior occipital gyrus and lingual gyrus for reading tasks. These results support that these areas are necessary for reading proficiency. Because this analysis controls the types of comprehension against each other, we cannot say that these are the only regions necessary for reading comprehension, or even that lesions within these areas will necessarily result in reading comprehension deficits. These results, however, can help us understand which patients are more likely to have auditory versus reading comprehension deficits.

## Limitations

The current study has some limitations. First, pre-morbid reading ability was not available. While none of the subjects had a history of developmental delay, the lack of more fine-grained pre-morbid reading levels precludes further interpretation regarding which results reflect the effects of injury versus reading abilities. Second, alexia subtype was not assessed since we did not use formal measures of post-stroke reading ability, except for the WAB-R supplement. This lack of more in-depth measures may have limited the ability of the study to provide a comprehensive analysis of reading abilities and does not allow for analysis that includes alexia subtype. Patients with severely limited speech output were excluded from this analysis that may also limit the generalizability of these results to persons with more severe aphasia. As such, the results of this analysis should be considered with caution when considering patients with severely limited speech output.

Additionally, A-SC requires a verbal response while R-SC prompts the answer in a multiple-choice format. As such, failure to respond accurately on the A-SC subtest may be due to word-finding or apraxia issues as opposed to pure comprehension. All four subtests contained an unequal number of questions and potential scores. In particular, the R-FC subtest contains a measure of accurate oral reading within the total score that cannot be dissociated from the comprehension aspects in this retrospective analysis without access to item level data. The limitations of the WAB-R subtests reduce the sensitivity of the analysis.

## Conclusions

Auditory comprehension strongly predicts reading comprehension impairments, but it cannot fully explain the variance. The regions associated with worse reading comprehension were the inferior temporal gyrus, fusiform gyrus and posterior inferior temporal gyrus for sentence completion, and the inferior occipital gyrus, lingual gyrus and posterior thalamic radiation for following sequential commands. These results can help inform potential treatment predictors for chronic reading impairment after stroke.

## Data Availability

The data that support the findings of this study are available from the corresponding author, upon reasonable request.
